# Tumor Grafting Induces Changes of Gut Microbiota in Athymic Nude Mice in the Presence and Absence of Medicinal *Gynostemma* Saponins

**DOI:** 10.1371/journal.pone.0126807

**Published:** 2015-05-20

**Authors:** Lei Chen, William C. S. Tai, Manreetpal S. Brar, Frederick C. C. Leung, W. L. Wendy Hsiao

**Affiliations:** 1 State Key Laboratory of Quality Research in Chinese Medicine, Macau University of Science and Technology, Macau, China; 2 Center for Cancer & Inflammation Research, School of Chinese Medicine, Hong Kong Baptist University, Kowloon, Hong Kong, China; 3 School of Biological Sciences, The University of Hong Kong, Hong Kong, China; Charité, Campus Benjamin Franklin, GERMANY

## Abstract

Recent findings have revealed that gut microbiota plays a substantial role in modulating diseases such as autism, rheumatoid arthritis, allergies, and cancer that occur at sites distant to the gut. Athymic nude mice have been employed for tumorigenic research for decades; however, the relationships between the gut microbiome and host’s response in drug treatment to the grafted tumors have not been explored. In this study, we analyzed the fecal microbiome of nonxenograft and xenograft nude mice treated with phytosaponins from a popular medicinal plant, *Gynostemma pentaphyllum* (Gp). Analysis of enterobacterial repetitive intergenic consensus (ERIC)-PCR data showed that the microbiota profile of xenograft mice departed from that of the nonxenograft mice. After ten days of treatment with Gp saponins (GpS), the microbiota of the treated mice was closer to the microbiota at Day 0 before the implantation of the tumor. Data obtained from 16S pyrosequencing of fecal samples reiterates the differences in microbiome between the nonxenograft and xenograft mice. GpS markedly increased the relative abundance of *Clostridium cocleatum* and *Bacteroides acidifaciens*, for which the beneficial effects on the host have been well documented. This study, for the first time, characterizes the properties of gut microbiome in nude mice responding to tumor implant and drug treatment. We also demonstrate that dietary saponins such as GpS can potentially regulate the gut microbial ecosystem by increasing the number of symbionts. Interestingly, this regulation of the gut ecosystem might, at least in part, be responsible for or contribute to the anticancer effect of GpS.

## Introduction

Accumulated evidence shows that the efficacy of drug treatments varies greatly from individual to individual, possibly influenced by the genetic polymorphism of individuals. It is also well recognized that factors such as nutrition, age, overall health, and gut bacterial distribution, influence the drug metabolism of an individual. Normal gut microbes make significant contributions to the overall health of their host including protection against potentially harmful microorganisms and stimulation of the immune system. Recent findings have revealed that gut microbes play an even greater role in modulating human metabolic phenotypes and individuals’ drug responses. On the one hand, the host’s dietary and drug uptake can alter the gut microbiota composition [1–3]. Conversely, microbes can influence the bioavailability and bioactivity of ingested products, including functional foods and herbal medicines [4, 5] as well as the host’s metabolic phenotypes [6]. Strikingly, accumulating evidence shows that the gut microbiota can influence diseases found in organs or tissues distant from the gut, such as obesity [2, 3], diabetes [7, 8], autism [9, 10], rheumatoid arthritis [11], allergies [12, 13], and chronic liver disease [14].

Recent studies have presented strong evidence showing that intestinal microbiota modulates the efficacy of chemotherapy. For example, it was found that beta-glucuronidase in intestinal microbiota contributes to the delayed diarrhea caused by the antitumor camptothecin derivative irinotecan hydrochloride (CPT-11) [15, 16]. Later study showed that mice tolerated higher dosages of CPT-11 when the drug was administered together with a beta-glucuronidase inhibitor [17]. Commensal intestinal microbiota might also play a prominent role in the development and severity of chemotherapy-induced mucositis in cancer patients [18, 19]. Animal studies have demonstrated that intestinal microbiota modulates the antitumor efficacy of cyclophosphamide, in part, through the induction of “pathogenic” Th17 cells [20]. Along this line, a recent report has shown that disruption of commensal microbiota impairs the response of subcutaneous tumors to CpG-oligonucleotide immunotherapy and platinum chemotherapy [21]. In addition to their impact on chemotherapy, the gut microbiota has been suggested to mediate the effect of diet or dietary compounds in cancer prevention and cancer risk. For instance, a resveratrol-supplemented diet significantly reduced the colonic tumors in rats through the reduction of bacterial glucuronidase [22]. A combination of beneficial microbes and specific dietary compounds has been found to prevent the development of colonic polyps in both animals and humans [23–25]. Overall, the dynamic interplay between the gut microbiota and ingested drugs or dietary compounds impacts on cancer risks and their treatment.

Athymic nude mice have been a major animal model for cancer research. Their impaired immune system facilitates tumor grafting avoiding the risk of graft rejection by the animal’s immune system. Early study of mouse gastrointestinal microecology showed that the loss of T-cell function does not drastically alter the cultivable gut microbiota in Balb/c athymic (nu/nu) mice as compared to heterozygous (nu/+) or thymus-implanted nude mice [26]. However, the impact of grafted tumors on the gut microbiota in nude mice and how gut microbiome in turn influence the host’s response to drug treatments have not been explored.

Saponins are found in many herbal and edible plants. *Gynostemma pentaphyllum* (Gp) has been consumed as an herbal tea and used as a folk medicine dating back to the sixteenth century, according to the Chinese *Compendium of Materia Medica*, where it is recommended for the treatment of various diseases, including cancer. The main active components in Gp are triterpenoid saponins [27]. In this study, we first demonstrate that GpS exert anticancer effects on tumor-bearing nude mice. Using ERIC-PCR and 16S pyrosequencing methods, we compared the gut microbiota compositions of nonxenograft and tumor-bearing xenograft nude mice, and investigated how GpS treatment could influence the composition of gut microbiota in healthy and tumor-bearing nude mice.

## Materials and Methods

### Cell line and culture medium

Rat 6 (R6) cell line is a subclone of Fisher rat embryo fibroblasts derived from the primary rat embryo cells in Freeman’s Laboratory [28]. The R6/GFP-*ras* cell line is a transformed clonal cell line established from Rat 6 fibroblast cultures transfected by a GFP-tagged *ras* oncogene in our laboratory [29]. Cells were grown in Dulbecco modified Eagle medium (DMEM) supplemented with 10% calf serum (Gibco, USA). Cultures were maintained in a humidified incubator at 37°C with 5% CO_2_ in air and incubated twice a week with fresh medium.

### Animals and treatments

Experimental procedures were conducted according to guidelines for the care and use of laboratory animals. All procedures were approved by the Baptist University Ethics Review Committee for Animal Research. Athymic nude mice (BALB/c-nu/nu) were purchased from the Chinese University of Hong Kong and maintained in individually ventilated cages (IVC) on a 12-h light/12-h dark cycle, 20–22°C temperature and 40–60% humidity with free access to food and water. Mice were fed with PicoLab Rodent Diet 20–5053 (LabDiet, USA). Xenograft was performed by injecting 10^6^ R6/GFP-*ras* transformed cells into the flank of each 7- to 8-wk-old mouse. The control mice were injected with a volume of PBS solution equivalent to the tumor volume. Tumors were blind-measured using an electronic caliper daily, and tumor volume was calculated using the formula (length × width^2^)/2. GpS extracted from the aerial parts of *Gynostemma pentaphyllum* was purchased from Hauduo Natural Products (Guangzhou, China). According to procedures outlined by Wu *et al*. [30], each batch of GpS was authenticated and chemically profiled (S1 Fig). GpS was dissolved in 0.5% carboxymethyl cellulose (CMC) at 50 mg/ml. A single dose of GpS at 750 mg/kg or solvent control was administered daily by gavage and the treatment was initiated the second day after implanting the GFP-*ras* cells and administered for 12 d. Six nonxenograft and six xenograft nude mice were used for investigating the impact of tumor implant on the gut microbiota. When determining the effects of GpS on the gut microbiota, six animals each were used for both nonxenograft-control and nonxenograft-GpS groups; while seven animals each were employed for xenograft-control and xenograft-GpS groups. Euthanasia of animals was carried out according to the guidance of the American Veterinary Medical Association (AVMA). Total 38 athymic nude mice were used in the experiment, and carbon dioxide (CO_2_) inhalation was used for euthanasia of mice.

### Fecal sample collection and bacterial genomic DNA extraction

Animal feces were collected from each mouse for two consecutive hours from 8:00 to 10:00 A.M. on day 0 (before the xenograft), and day 5 and day 10 after GpS treatment. All fecal samples were immediately stored at -20°C for later DNA extraction. Total genomic DNA was isolated from fecal samples as described with a slight modification [31, 32]. In brief, fecal samples of a weight of 0.1 g were vortexed in 4 ml of sterile PBS (pH 7.4) for 5 min, and then centrifuged at 40×g for 8 min to collect the upper phase containing the bacteria. After repeating this procedure once, the supernatant was centrifuged at 2000×g for 8 min. The supernatant was discarded and the bacterial pellets were washed twice with PBS to isolate the DNA. DNA concentration was determined using NanoDrop 1000 spectrophotometry.

### ERIC-PCR

ERIC sequences are noncoding, highly conserved intergenic repeated sequences that reside in the genomes of various bacterial species, including enterobacteria [33, 34]. ERIC-PCR was used to profile gut microbiomes [35] by using fecal genomic DNA as the template and a pair of ERIC specific primer sequences: ERIC 1R (5’-ATGTAAGCTCCTGGGGATTCAC-3’) and ERIC 2 (5’-AAGTAAGTGACTGGGGTGAGCG-3’). The PCR reaction was optimized and determined using an orthogonal array design. The 25 μl reaction mixture consisted of 5 μl 5×PCR reaction buffer, 250 μM dNTP, 2 mM Mg^2+^, 0.4 μM primers, 1.5 units Hotstart Taq polymerase, and 50 ng fecal genomic DNA. PCR was performed as follows: 94°C for 5 min, followed by 35 cycles of 95°C for 50 s, 49°C for 30 s, 46°C for 30 s, 72°C for 3 min, and then a final extension at 72°C for 9 min [36]. 10 μl of each PCR product was loaded on 2% (w/v) agarose gels containing 0.5 μg/ml ethidium bromide and run for 40 min at 100 V. A DNA ladder (0.1–10.0 kb) was used as the DNA size marker (NEB, N3200). Agarose gels were photographed using the Gel Doc XR+ system.

### Data analysis of ERIC-PCR fingerprints

Partial least squares discriminant analysis (PLS-DA) was performed to visualize the dynamic changes of microbiota composition before and after treatments. The banding patterns of ERIC-PCR products were digitized by Image Lab 3.0 system (Bio-Rad) to generate the data based on the sum of the distance and the intensity of each DNA band within each sample lane. The scores were subjected to PLS-DA plot using SIMCA-P 12.0 tool (Umetrics, Umea, Sweden) for which the confidence level was set at 95% (P<0.05). The correlation coefficient was calculated and used to assess the correlation between the two samples applying the CORREL function in Microsoft Office Excel 2003.

### 16S rRNA gene pyrosequencing of fecal DNA samples

Three fecal samples randomly picked from each experimental group on Day 10 were subjected to further analysis by using 16S rRNA gene pyrosequencing. Pyrosequencing was conducted on the 454 Sequencer (Junior System) (Life Sciences Corp., USA). To begin, PCR was performed on each sample in a final reaction volume of 25 μl consisting of 0.1–2 μl DNA, 300 nM of each primer (563F and 1064R of the 16S rRNA gene), 2.5 μl 10x Expand High Fidelity buffer (Roche), 200 μM PCR Grade Nucleotide Mix, and 2.6 units of the Expand High Fidelity Enzyme mix (Roche). The reaction volume was adjusted using Milli-Q H_2_O. The forward primer of each reaction was assigned a unique 11-nt barcode, then PCR was conducted with an initial denaturation at 94°C for 2 min, followed by 35 cycles of 94°C for 15 s, 58°C for 20 s, and 72°C for 1 min. An elongation reaction for 7 min at 72°C was finally performed, followed by cooling at 4°C until collection. Amplicon sizes were confirmed on 1% agarose gel and purified using the PureLink Quick Gel Extraction Kit (Life Technologies). Amplicon libraries were quantified using the Quant-iT PicoGreen dsDNA Assay Kit (Life Technologies) with a FLUOstar OPTIMA F fluorometer (BMG Labtech GmbH, Offenburg, Germany), and visually assessed using the FlashGel System (Lonza Group Ltd., Basel, Switzerland). Emulsion-PCR and pyrosequencing using titanium chemistry on the GS Junior System (454 Life Sciences Corp., Branford, CT, USA) was conducted according to manufacturer instructions.

### Denoising and analysis of pyrosequencing data

The Quantitative Insights into Microbial Ecology (QIIME) software package version 5.0 was used to process and analyze the raw pyrosequencing data [37]. Pyrosequencing data was first processed using QIIME and filtered for quality by removing sequences that were shorter than 200bp or longer than 1kb with a quality score below 25, or that contained primer mismatches, uncorrectable barcodes, or had a homopolymer run, or had more than 6 ambiguous bases. The operational taxonomic units (OTUs) in QIIME are clusters of sequences; these are used to represent taxonomic relatedness. Denoising raw sequences was done to reduce the number of OTUs. Sequences from all samples were clustered into OTUs based on their similarity. The denoised sequences were assigned to OTUs with a 97% identity threshold, and the most abundant sequence from each OTU was selected as a representative sequence. Taxonomy was assigned to the OTUs by using the Basic Local Alignment Search Tool (BLAST). For tree-based analyses, PyNAST was used to align the representative sequences of each OTU, and the FastTree algorithm was used to build a phylogenetic tree [38]. The differences in overall microbiota composition between compared samples were determined using the unweighted UniFrac metric. The raw 454 pyrosequencing data were deposited in NCBI's Sequence Read Archive (SRA) database under accession number of SRP052560.

The linear discriminant analysis (LDA) effect size (LEfSe) method [39], which is an algorithm for high-dimensional class comparisons with a particular focus on metagenomic analysis, was further applied to evaluate the key phylotypes responsible for the differences between microbial communities by using the processed data from QIIME. LEfSe uses LDA scores to estimate the effect size of each differentially abundant feature, and to rank the relative difference of microbial taxa that are discriminative with biological consistency and statistical significance. The alpha value used for the algorithm of LEfSe was internally set at 0.05, which corresponded to 95% confidence level (P<0.05) to detect features with significant differential abundance and to test the biological consistency. The OTU network was generated using QIIME and was visualized using Cytoscape. The Shannon-Wiener diversity index (H') was used to evaluate the diversity of the microbial communities. A Venn diagram was used to depict the unique and shared taxa among microbial communities.

### Statistical analysis

The data are presented as mean ± SEM, and statistical comparisons were performed using one-way ANOVA followed by Dunnett’s post test with the GraphPad Prism version 5.00 (GraphPad Software, San Diego, CA, USA) or Student’s t-test at P values of <0.01(**) or <0.05(*).

## Results

### Significant alteration in the gut microbiota of xenograft nude mice

**Fig 1:**
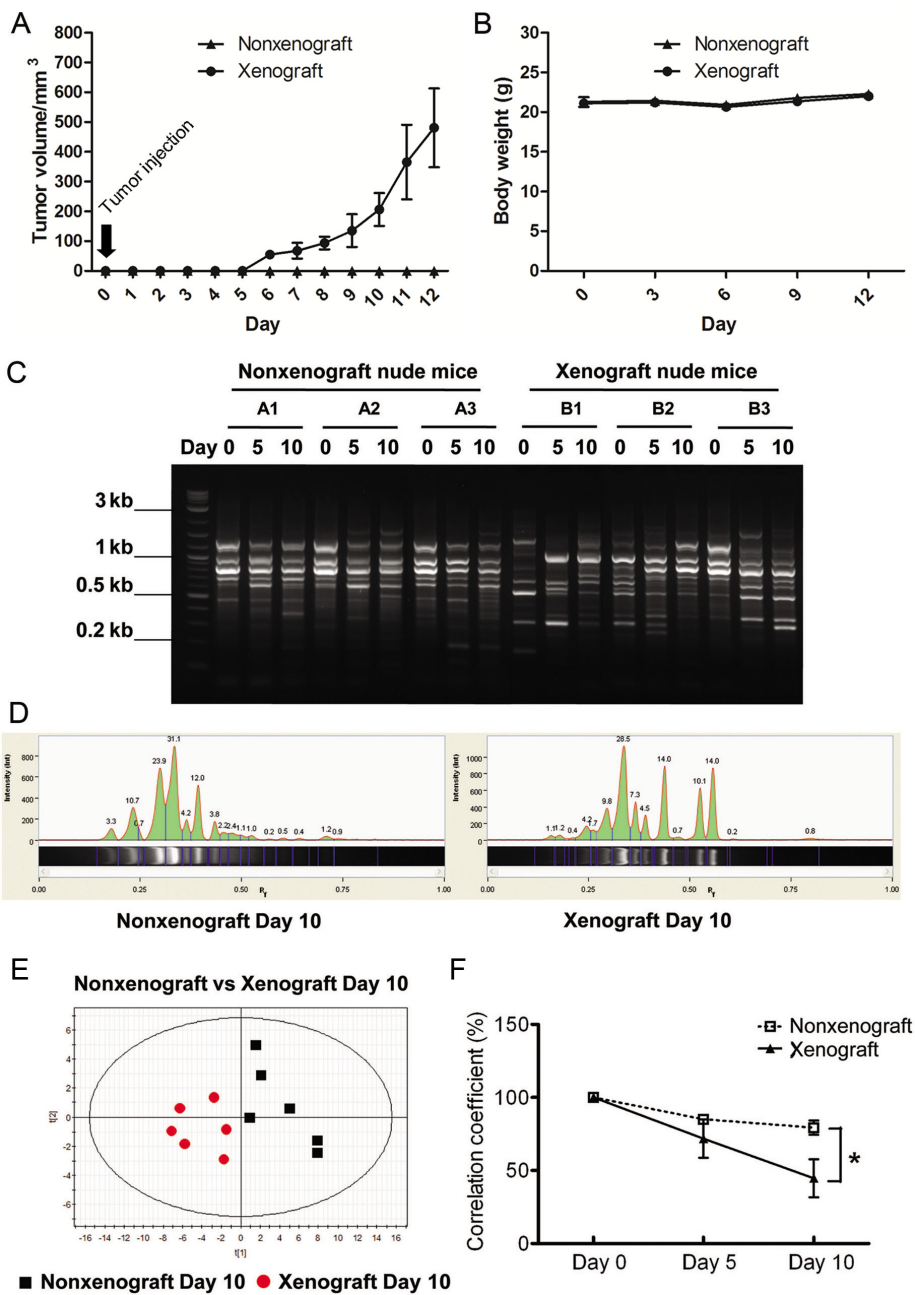
Comparison of fecal microbiota between nonxenograft and xenograft nude mice. (A) Tumor growth curve. (B) Body wight. (C) Representative ERIC-PCR DNA fingerprints of the fecal microbiota of individual nonxenograft and xenograft nude mice. Fecal samples were collected before xenograft (Day 0), and 5 & 10 d upon saline or tumor cells injection. (D) Digitization of ERIC-PCR DNA fingerprints. Gel images were digitized by Image Lab 3.0 system (Bio-Rad). The peak area corresponded to the intensity of ERIC-PCR band shown in the gel image. (E) PLS-DA plot of ERIC-PCR data from fecal microbiota of nonxenograft and xenograft nude mice at Day 10. Black square: nonxenograft nude mice; Red dot: xenograft nude mice. (F) Correlation coefficients of fecal microbiota of nonxenograft and xenograft nude mice. All data were expressed as the percentage change over the Day 0 control of each mouse, and presented as the mean ± SEM (* P < 0.05, ** P < 0.01 versus nonxenograft group, n = 6/group).

To investigate whether a tumor implanted in the flank of a mouse would affect microbiota in the gut, fecal samples were collected from mice at Days 0 (before tumor cells or saline injection), 5 and 10. Meanwhile, the tumor volume and body weight of mice were recorded during the entire experimental period (Fig 1A and 1B). The fecal microbial profiles were analyzed using ERIC-PCR. The banding patterns of ERIC-PCR (Fig 1C) were then digitized using the Image Lab 3.0 system (Bio-Rad) and analyzed using PLS-DA analysis (Fig 1D). Although inter-animal variation in microbial profile appeared at Day 0, a clear separation between the samples from Day 10 normal and tumor-bearing mice appears in the PLS-DA plot (Fig 1E) and in the correlation coefficient plot (Fig 1F). These findings suggest that the xenografted tumors caused alteration of gut microbiota composition as compared to the nonxenograft mice.

### GpS inhibited tumor growth in nude mice

**Fig 2:**
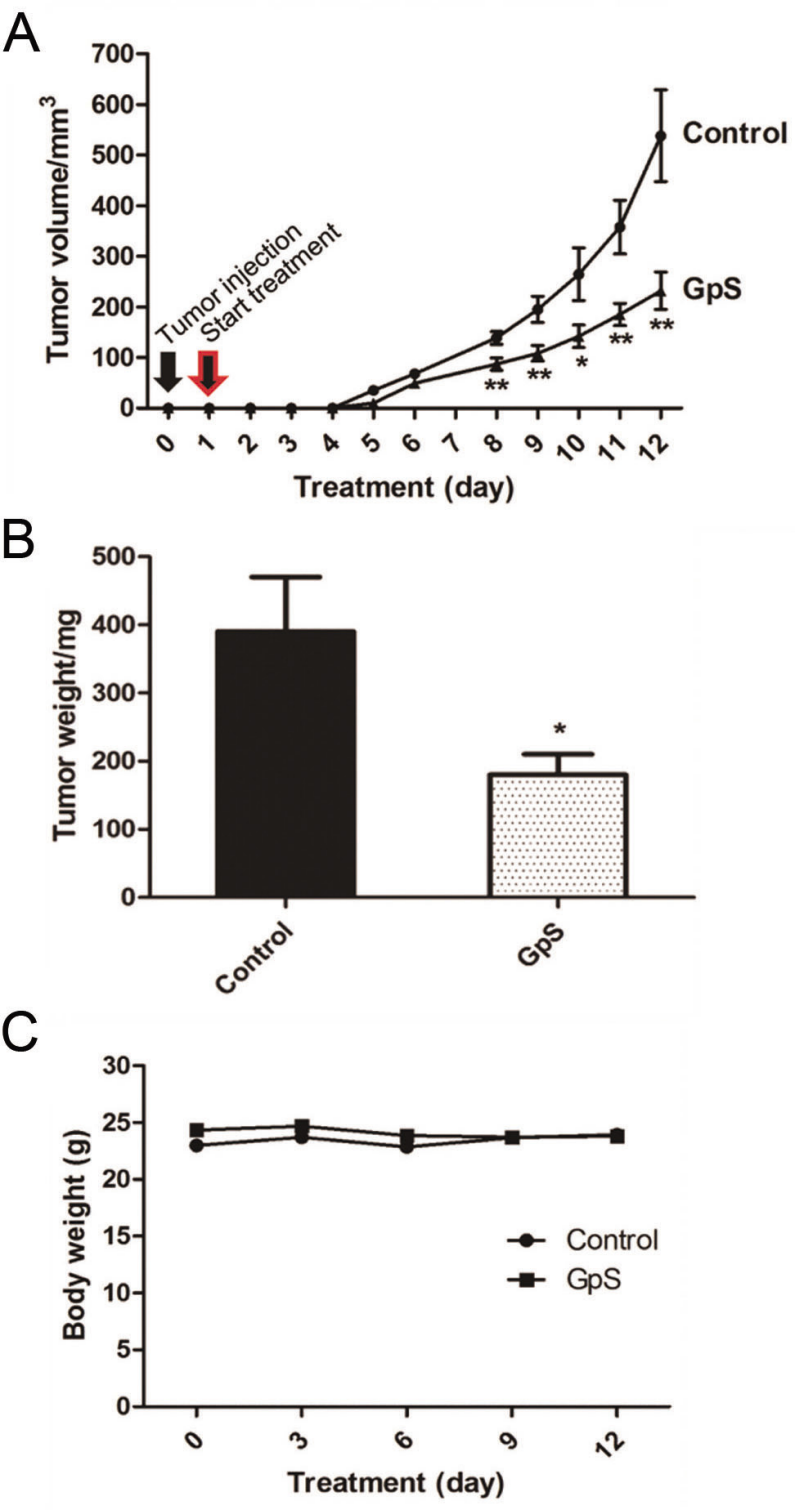
Effect of GpS on tumor growth in nude mice. (A) Tumor volume, (B) Tumor weight and (C) Body weight. Xenografting was performed by injecting 10^6^ R6/GFP-*ras* transformed cells into the flank of each 7- to 8-wk-old mouse. A single dose of GpS at 750 mg/kg or solvent control was administered daily by gavage and the feeding was initiated the second day after implanting the GFP-*ras* cells. All data are presented as the mean ± SEM (* P < 0.05, ** P < 0.01 versus control) (n = 7/group).

In our previous study, GpS demonstrated strong anti-cancer effect against the growth of R6/GPS-*ras* transformed cells at nontoxic dosages (unpublished data). To assess the effect of GpS on the xenografted tumors, parallel sets of nonxenograft and xenograft nude mice were given single daily doses of GpS (750 mg/kg) by gavage, initiated on the second day after implant of the transformed R6/GFP-*ras* cells. The tumor volume and tumor weight of the GpS-treated group were 60% and 50%, respectively, lower than the untreated group (Fig 2A and 2B). No weight loss was observed in the experimental animals (Fig 2C). There was no observable damage of internal organs of the treated animals. These results echo the previous report on the chronic toxicity studies of Gp where the rodents were treated 750 to 1000 mg/kg for long duration of time [40, 41]. From these GpS-treated mice, fecal samples were collected for subsequent analysis and comparison with the non-treatment groups.

### GpS treatment induced unique alteration of microbiota in xenograft, but not in nonxenograft mice

**Fig 3:**
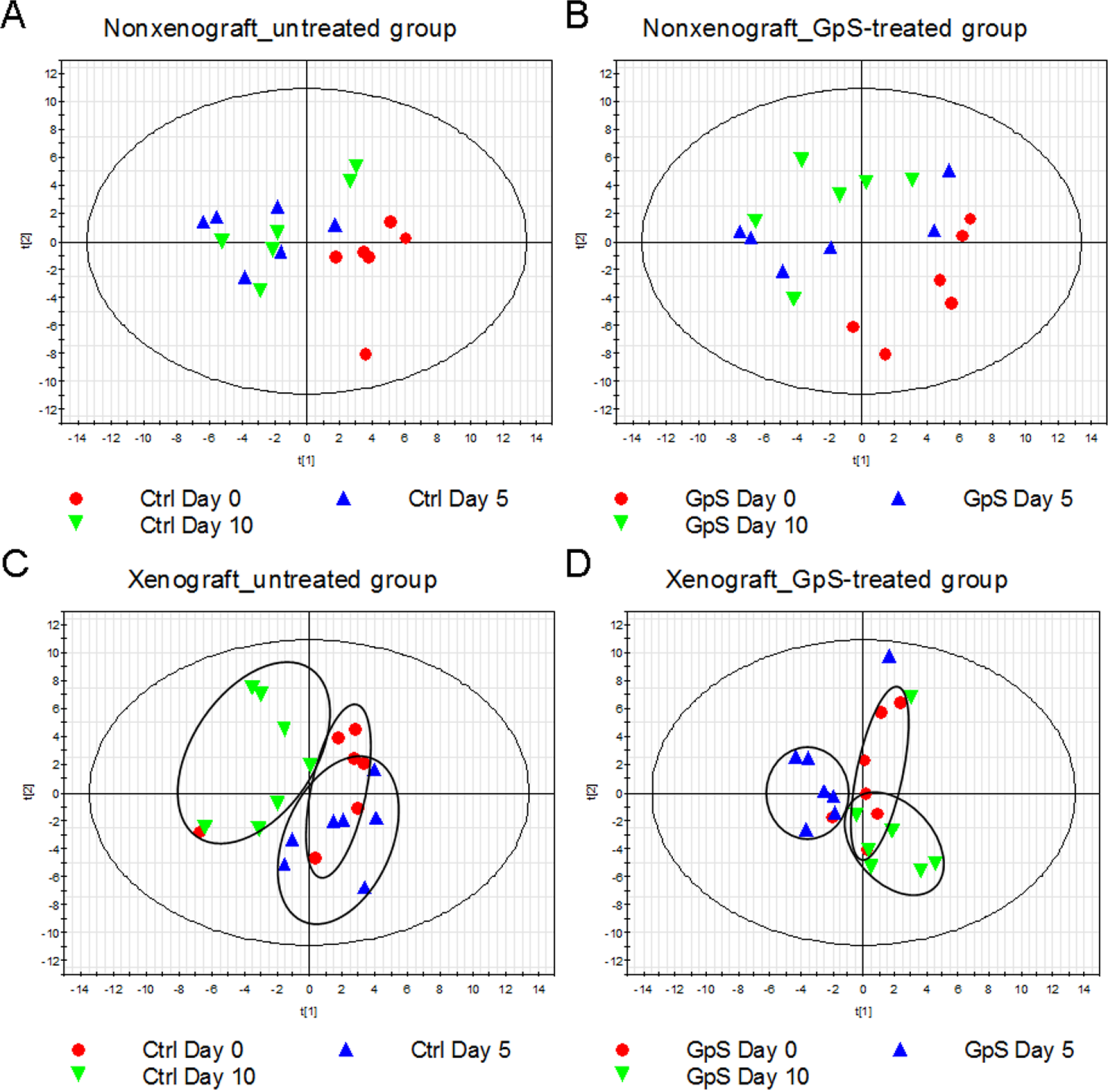
ERIC-PCR analysis of the effect of GpS on fecal microbial composition of nude mice. PLS-DA plots of (A) untreated nonxenograft mice, (B) GpS-treated nonxenograft mice, (C) untreated xenograft mice, and (D) GpS-treated xenograft mice.

Fecal samples collected from the above four experimental groups—nonxenograft and xenograft nude mice with and without GpS treatment—at Days 0, 5, and 10 were subjected to ERIC-PCR analysis. The PLS-DA plots, based on the ERIC-PCR banding patterns of the fecal DNA samples, showed no clear difference between samples collected in the presence or absence of GpS in nonxenograft mice (Fig 3A and 3B). In the xenograft mice, by contrast, clusters of samples collected from different time points were observed, where Day 0 samples were mainly located in the right quadrant (Fig 3C and 3D). Intriguingly, after treatment, the Day 10 samples from the GpS-treated xenograft mice appeared in the right lower quadrant overlapping with Day 0 group (Fig 3D). On the other hand, the samples collected from the untreated mice gathered in the left quadrant away from the Day 0 control (Fig 3C).

### 16S pyrosequencing further revealed marked differences between nonxenograft and xenograft nude mice in microbial communities

To further examine the detailed composition of the fecal microbiome of the experimental animals, we randomly selected three fecal DNA samples collected on Day 10 from each of the four experimental groups, namely the nonexenograft mice with and without GpS treatment; and the xenograft mice with and without GpS treatment, and performed 16S rRNA gene pyrosequencing. A total of 147,128 reads passing quality control were produced in this study, with an average of 12,261 sequences per sample. A total of 399 distinct OTUs were determined after denoising using QIIME [37].

**Fig 4:**
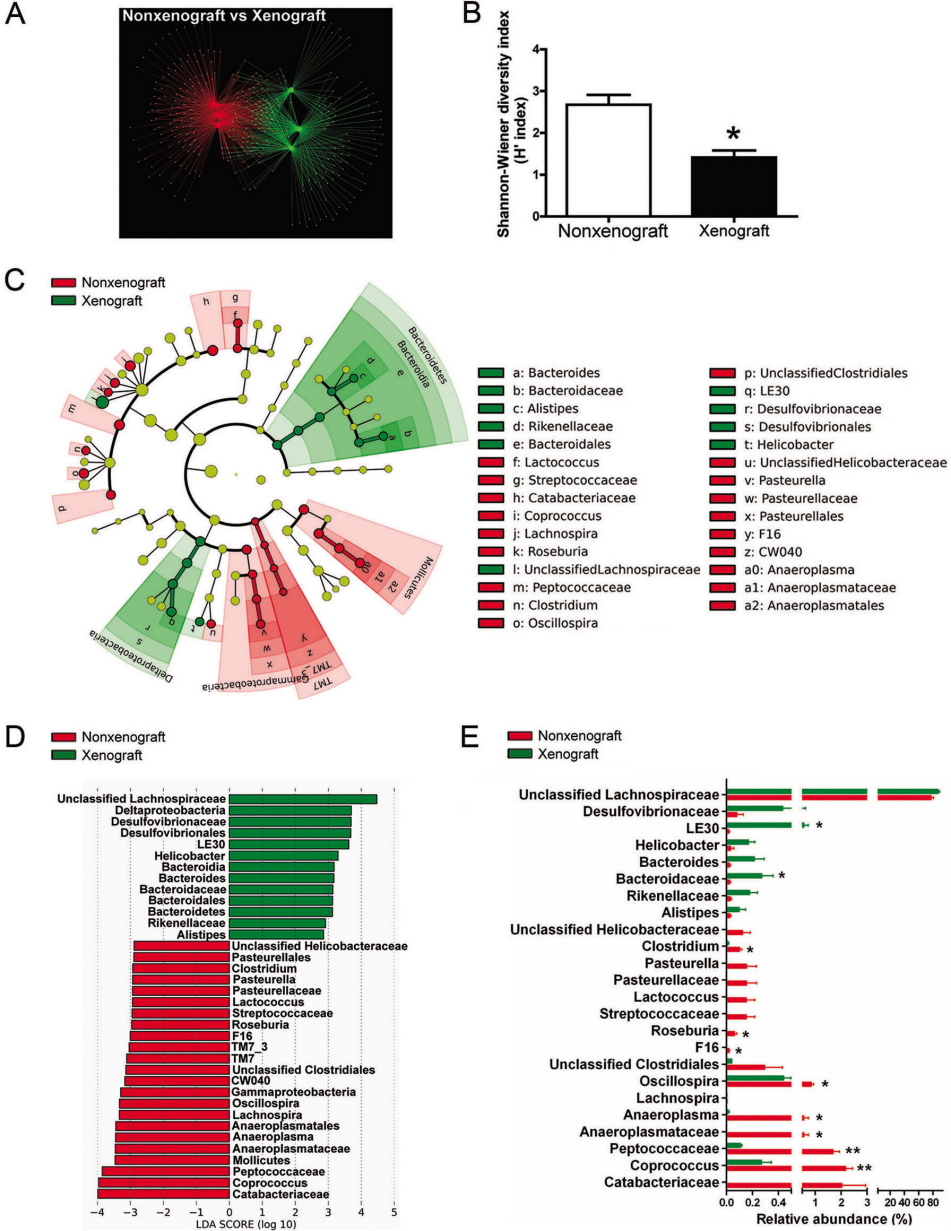
Pyrosequencing analysis of fecal microbiota of nonxenograft and xenograft nude mice. (A) Comparisons of overall OTU networks of fecal microbiota between the nonxenograft (red) and xenograft (green) mice. (B) Shannon-Wiener diversity index (H'). The analysis is based on the percentage of classified OTUs of each sample. H' index was used to calculate the diversity of microbial communities. H' = ∑ - (Pi * ln Pi). (C) Taxonomic representations of fecal microbiome of nonxenograft and xenograft nude mice. The differentially abundant taxa are presented with designated colors using LEfSe method. The taxa from nonxenograft and xenograft mice are colored in red and green, respectively. The taxa with nonsignificant changes between the nonxenograft and xenograft mice are colored in yellow. Each small circle’s diameter represents the taxon abundance. (D) Histogram of the LDA scores of fecal 16S rRNA gene sequences of nonxenograft (red color) and xenograft (green color) mice. LDA scores characterized the magnitude of differential abundance in the microbial taxa between compared samples. (E) The relative abundance (%) of differentially abundant families and genera. Nonxenograft nude mice, n = 3; Xenograft nude mice, n = 3. Data are presented as the mean ± SEM (* P < 0.05, ** P < 0.01, nonxenograft versus xenograft group).

We first compared the fecal microbiome of the nonxenograft and xenograft mice without the influence of GpS. The OTU network analysis clearly separated the nonxenograft from the xenograft mice, suggesting that tumor growth was the likely cause of the differences in fecal microbiota composition (Fig 4A). Nonxenograft and xenograft mice had 112 and 91 unique OTUs, respectively, and shared 110 OTUs. Xenograft mice exhibited reduced microbial diversity compared with nonxenograft mice based on the calculated Shannon-Wiener diversity index (Fig 4B).

The 16S pyrosequencing data were analyzed using LEfSe to identify the key phylotypes responsible for the differences in fecal microbiota composition between the nonxenograft and xenograft mice. As shown in Fig 4C, the taxonomic distribution of fecal microbiota between the two groups varied significantly at all taxonomic levels. At the phylum level, the most differentially abundant bacterial taxon in the feces of nonxenograft mice was *TM7*, whereas *Bacteroidetes* was overrepresented in xenograft mice. Compared with the nonxenograft mice, the xenograft mice had relatively high levels of *Deltaproteobacteria*, but low levels of *Gammaproteobacteria*, both of which are Gram-negative proteobacteria. *Mollicutes*, in the phylum *Tenericutes*, were also underrepresented in xenograft mice. The histogram of the LDA scores (Fig 4D) and the relative abundance scores (Fig 4E) further illustrated a clear difference between the nonxenograft and xenograft mice in the composition of biologically clades. Several clades in the phylum *Firmicutes*, such as *Catabacteriaceae*, *Peptococcaceae*, and *Coprococcus*, were particularly abundant in nonxenograft but not in xenograft mice (Fig 4E).

### Xenograft and nonxenograft mice responded differently to GpS treatment

**Fig 5:**
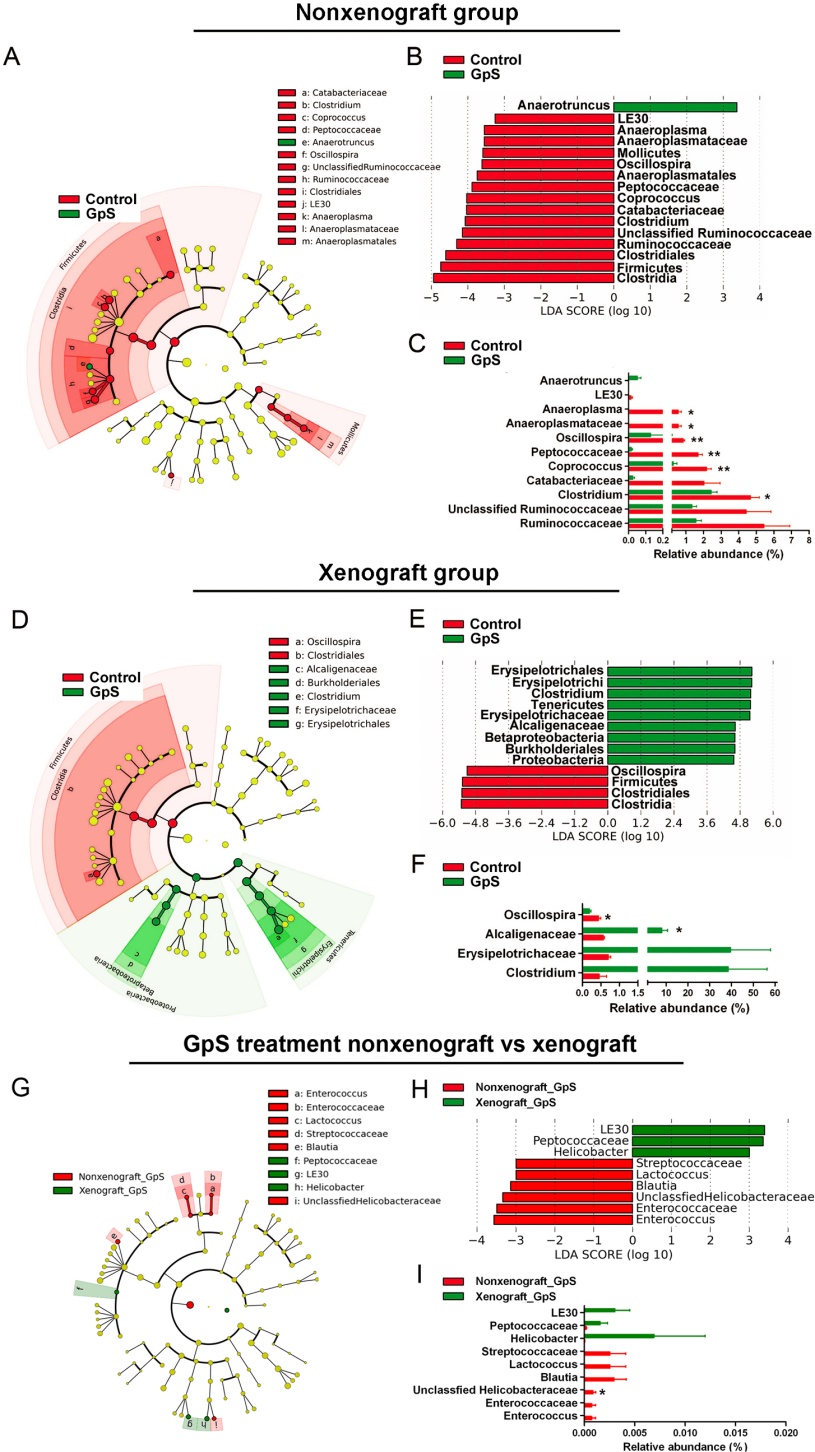
LEfSe analysis of pyrosequencing data obtained from different experimental groups. Effects of GpS on fecal microbiome in nonxenograft (A-C) and xenograft (D-F) nude mice. Data are presented as the mean ± SEM (* P < 0.05, ** P < 0.01, versus control group, n = 3/group). (G) to (I): comparison of differentially abundant bacterial populations between the nonxenograft and xenograft groups treated with GpS, data are presented as the mean ± SEM (* P < 0.05, xenograft versus nonxenograft group, n = 3/group). (A), (D), & (G): taxonomic representations of fecal microbiome. (B), (E), & (H): histograms of the LDA scores for differentially abundant clades. (C), (F), & (I): the relative abundance of differentially abundant families and genera.

To view in detail the impact of GpS treatment on gut microbiota, we took the 16S pyrosequencing data from the above four experimental groups and analyzed the phylogenic compositions of fecal samples using LEfSe. The taxonomic data are displayed as cladograms in Fig 5. In the nonxenograft group, the classes of *Clostridia* and *Mollicutes* were found to be particularly differentially abundant (Fig 5A). Within *Clostridia*, families such as *Catabacteriaceae*, *Peptococcaceae*, and *Ruminococcaceae*, and genera such as *Clostridium*, *Coprococcus*, and *Oscillospira*, were all found to be more abundant in the untreated nonxenograft mice than in the GpS-treated mice. Within the class *Mollicutes* in the phylum *Tenericutes*, lineages including *Anaeroplasmatales*, *Anaeroplasmataceae*, and *Anaeroplasma* were the prevalent clades in the nonxenograft group without GpS treatment (Fig 5B). *Anaerotruncus*, a genus in *Clostridia*, was the only differentially abundant taxon detected in the treated mice (Fig 5C).

In the xenograft mice, three dominant phyla showed differential responses to GpS treatment: *Firmicutes*, *Proteobacteria*, and *Tenericutes*. Within these phyla, *Clostridia*, *Betaproteobacteria*, and *Erysipelotrichi* were the predominant classes, respectively. The control and GpS-treated mice exhibited different portions of *Clostridia* (95.84% vs. 49.08%), *Betaproteobacteria* (0.81% vs. 8.01%) and *Erysipelotrichi* (1.65% vs. 39.58%) (Fig 5D). Differences in bacterial community structure between the control and GpS-treated mice were also observed. In the GpS-treated xenograft mice, two lineages were notably high in relative abundance compared with the controls: the *Proteobacteria*-*Betaproteobacteria*-*Burkholderiales-Alcaligenaceae* lineage and the *Tenericutes*-*Erysipelotrichi*-*Erysipelotrichales*-*Erysipelotrichaceae*-*Clostridium* lineage (Fig 5D). At the family level of these two particular lineages, GpS-treated xenograft mice showed higher levels of *Alcaligenaceae* (8.01% vs. 0.81%) and *Erysipelotrichaceae* (39.58% vs. 1.65%) compared with the control. At the genus level, *Clostridium* presented the greatest difference in abundance between the two groups (Fig 5F). *Clostridium* constituted less than 0.5% of total bacteria in controls, but was more prevalent in GpS-treated mice (mean 38.48%). We then further compared the differentially abundant bacterial populations between the nonxenograft and xenograft groups treated with GpS (Fig 5G and 5I). However, the relative abundance of identified taxa was all less than 0.01%, indicating the differences were not so striking between the two groups.

**Fig 6:**
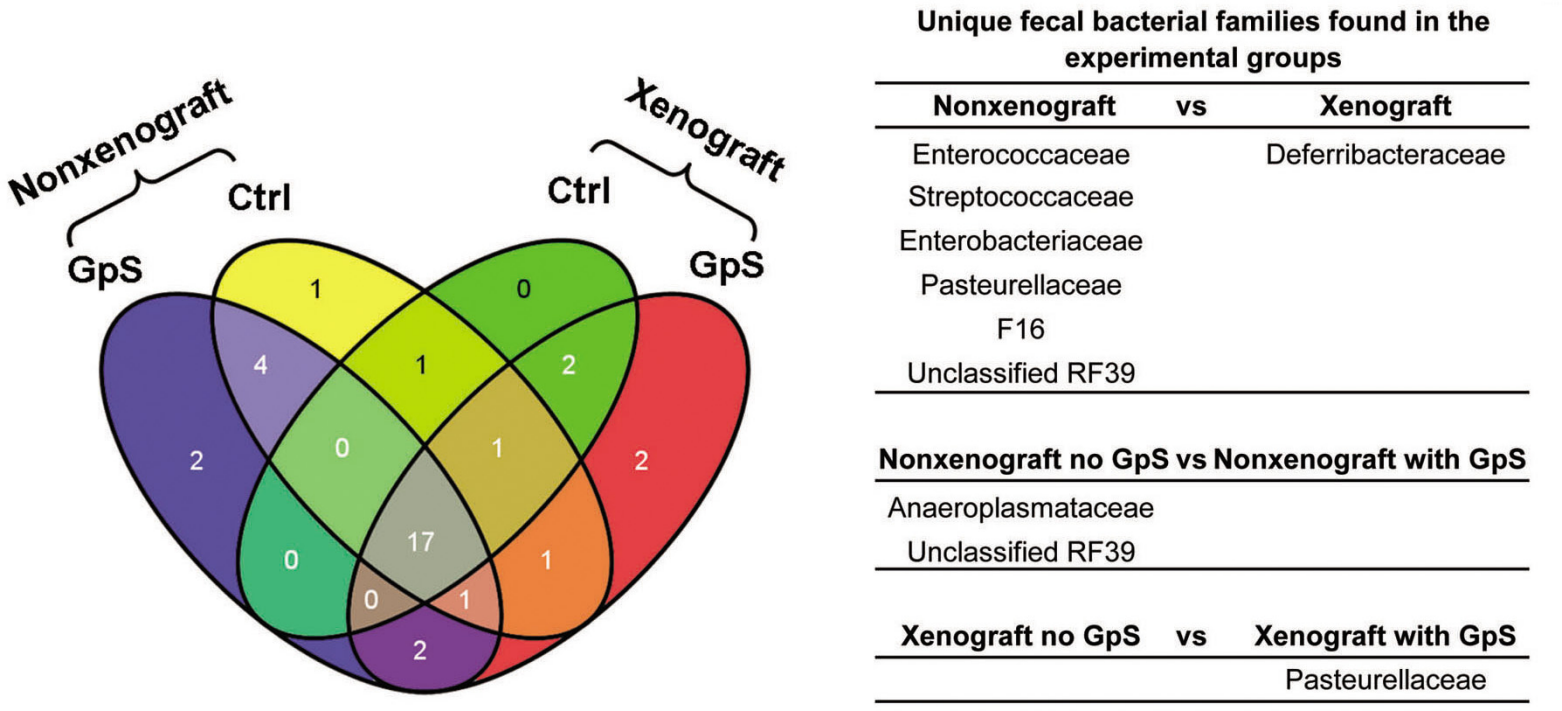
Unique fecal bacterial families identified from different treatment groups. Venn diagram showing the number of unique and shared bacterial families of nonxenograft and xenograft nude mice, with or without GpS treatment. Yellow: nonxenograft nude mice; Green: xenograft nude mice; Blue: nonxenograft nude mice with GpS treatment; Red: Xenograft nude mice with GpS treatment. The table indicates the unique families to each treatment group (n = 3/group).

To identify which taxa were unique to different treatment groups, we compared the bacterial families using the Venn diagram. As shown in Fig 6, there were seven and two unique bacterial families found in the nonxenograft and xenograft mice, respectively, whereas 19 bacterial families appeared in both groups. Comparing nonxenograft mice with and without GpS treatment, 22 bacterial families were shared, whereas the control and GpS-treated groups each had 4 unique bacterial families. By contrast, in xenograft mice, 20 bacterial families were found in both the control and GpS-treated mice. Only one unique bacterial family was found in the control whereas 6 unique families were found in the GpS-treated xenograft mice. These unique bacterial families with a mean relative abundance > 0.01% are listed in Fig 6.

### Two beneficial bacterial species were markedly enhanced by GpS treatment

**Fig 7:**
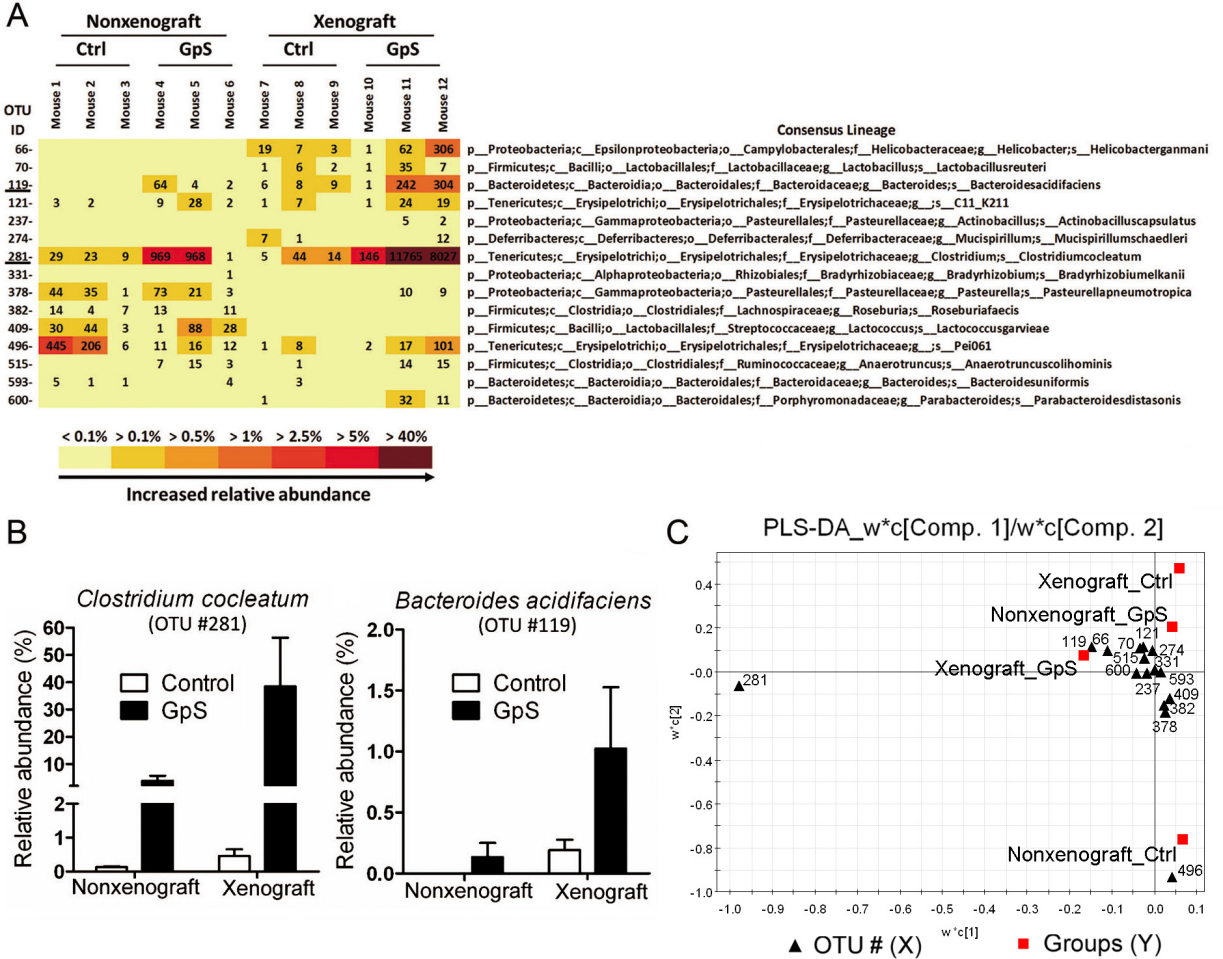
Differentially abundant bacterial species responding to GpS treatment. (A) OTU heatmap based on the 16S rRNA gene reads identified bacterial species. The figures displayed in the OTU heatmap are the raw OTU counts of individual mouse sample. p: phylum; c: class; o: order; f: family; g: genus; s: species. (B) The relative abundance of *Clostridium cocleatum* and *Bacteroides acidifaciens* in nonxenograft and xenograft nude mice with or without GpS treatment. Data are presented as the mean ± SEM (n = 3/group). (C) PLS-DA W*C loading plots. The data of relative abundance of identified bacterial species was subjected to PLS-DA and generate W*C loading plots by SIMCA-P software. The W*C loading plots was used to measure the degree of contribution of each identified OTU in the segregation of microbial composition upon treatment.

The consensus lineage map of OTUs generated by QIIME enabled bacterial identification at the genus level, and in some cases, at the species level. Two of the OTUs related to *Clostridium cocleatum* and *Bacteroides acidifaciens* were identified. In both xenograft and nonxenograft mice, these species increased in number following GpS treatment. GpS-treated nonxenograft mice showed 28 times higher levels of *C*. *cocleatum*, compared to the control mice. In the GpS-treated xenograft mice, *C*. *cocleatum* was elevated 80 times above those found in the control group. The relative abundance of *B*. *acidifaciens* increased five-fold compared with the untreated controls (Fig 7B). We further analyzed the data in a PLS-DA model and plotted the W*C loading plots using SIMCA-P, which measures the level of contribution of each variable in the segregation of microbial composition upon treatment. As shown in Fig 7C, OTU #281 (*C*. *cocleatum*) was farthest away from the axes, indicating its strongest level of contribution. The substantial increase of *C*. *cocleatum* appeared to be an important contributor of fecal bacterial community structures in GpS-treated tumor-bearing mice.

## Discussion

The athymic nude mouse is the animal model most often used for tumorigenic studies. While many aspects of the tumor-bearing mice’s physiology have been explored, the impact of the grafted tumors on the gut microbiota in nude mice and how gut microbiota would influence the drug responses of the hosts are unexplored. This study presents an investigation on how gut microbiota in nude mice is influenced by the xenografted tumor and by the dietary and medicinal saponins that exert anti-cancer effect under nontoxic dosages. ERIC-PCR and 16S pyrosequencing data demonstrated dynamic responses of intestinal microbial communities to tumor grafting in the presence or absence of GpS-treatment.

Early reports on the gastrointestinal microecology of Balb/c nude mice suggested that the lack of a thymus did not significantly affect the normal microbial flora of the mouse [26]. Studies also cited the presence of activated macrophages upon stimulations with exogenous factors, indicating that there might be intracellular components linking the tumor cells to the host flora through the host immune system [42]. In fact, gut microbiota is mobile. Alterations in anatomical location, in addition to the composition, of the intestinal commensal bacteria are associated with numerous chronic diseases [2, 43]. Significant alteration of intestinal microbial composition in tumor-xenografted mice treated with the anticancer agent cyclophosphamide has also been reported in a very recent study [20]; these results are consistent with our observation. Our results may indicate that tumor progression might lead to dysregulation of the host’s immune system, which in turn could account for the alteration in gut microbiota of the xenografted mice.

ERIC-PCR has been used to detect bacterial species in the *Enterobacteriaceae* and *Vibrionaceae* families, including well-known organisms such as *Escherichia coli*, *Salmonella enterica*, *Yersinia pestis*, and *Vibrio cholera*. ERIC primer sequences have also been found in the genome of various bacterial species [33, 34]. We observed that tumor growth rapidly altered the composition of gut microbiota (Fig 1). Intriguingly, as the tumor gradually shrank in response to GpS treatment, the microbiota more closely resembled populations in the guts of animals before implantation of the tumor cells (Fig 3D). This phenomenon was not observed in nonxenograft mice receiving the same GpS treatment. Such alterations in microbiota composition demonstrate a dynamic interaction of the gut microbes, the tumor-bearing host and the ingested phytosaponins. Whether this change was the result of tumor regression or it was the result of GpS treatment requires further investigation. Saponins exhibit many biological and pharmacological effects, however, one of the known traits of saponins is the poor bioavailability [44]. Whether part of effects of saponins lie on the interplay between the gut lumen and the microbiota is of interest to be explored in the future.

Fecal microbial communities were assessed using 16S pyrosequencing to obtain a comprehensive profile. At the phylum level, *Tenericutes*, *Proteobacteria*, and *Bacteroidetes* were more abundant in GpS-treated mice than in the controls, whereas *Firmicutes* showed the opposite pattern, being less abundant in GpS-treated mice, particularly in tumor-bearing mice. A shift in the ratio between *Firmicutes* and *Bacteroidetes* has been reported in many other studies, and such a shift has been linked to numerous diseases, such as obesity [2, 3]. The pyrosequencing data indicated that the *Bacteroidetes*/*Firmicutes* ratio showed an increasing trend after 10 d of GpS treatment in xenograft mice. GpS treatment also increased the relative abundance of *Proteobacteria*, the major group of Gram-negative bacteria in the gut. The lipopolysaccharide (LPS) in the outer layer of the Gram-negative bacteria is known to stimulate the immune system and has been recognized as a treatment for cancer [45]. The increased numbers of *Proteobacteria* in GpS-treated mice might enhance the secretion of LPS, thus activating an immune response against tumor growth, directly or indirectly.

The pyrosequencing analysis has identified several species of bacteria responding to GpS treatment as indicated in Fig 7A. Two species *C*. *cocleatum* and *B*. *acidifaciens*, which have several well-documented beneficial effects, were markedly increased in both nonxenograft and xenograft mice treated with GpS (Fig 7B and 7C). A previous study indicated that a strain of *C*. *cocleatum* protects against the colonization of the gut by the pathogenic *Clostridium difficile*, revealing multiple glucosidase activities that might degrade the oligosaccharide chains of mucin in the digestive tract [46]. *C*. *cocleatum* has been shown to be significantly decreased in irritable bowel syndrome patients [47, 48]. *C*. *cocleatum* also plays a role in the metabolism of diglucoside, and has a deglycosylation function [49]. *Clostridium* bacteria are a major component of mammalian gut microbiota and are responsible for promoting antiinflammatory immune responses. We speculate that *C*. *cocleatum*, which increased considerably in GpS-treated xenograft mice, is responsible for most of the differences between the GpS-treated xenograft mice and the controls. In addition to the above-mentioned well-documented beneficial effects, *C*. *cocleatum* may play a role similar to symbionts by taking part in the metabolism of Gp saponins by enhancing the glucosidase activities.

*B*. *acidifaciens* was first isolated from the cecum of mice [50]. *B*. *acidifaciens* and its close relative, *B*. *uniformis*, were found to be associated with the degradation of isoflavones in human feces [51]. A recent study demonstrated that *B*. *acidifaciens* promoted IgA production [52]. It is reasonable to postulate that the beneficial effects of *C*. *cocleatum* and *B*. *acidifaciens* support the anticancer effects of GpS. Changes in gut microbiota were more apparent in GpS-treated xenograft mice than in GpS-treated nonxenograft mice; that is, the pathological conditions of the xenograft enhanced the effects of GpS treatment. Besides *C*. *cocleatum* and *B*. *acidifaciens*, the change of OTU #496, an undefined species named Pei061, is also notable. However, the information of Pei061 is very limited based on current literature retrieval.

In summary, our results indicated that grafted tumors can significantly alter the composition of gut microbiota, indicating an active interaction between gut flora and the tumor-bearing host. We also demonstrated that GpS tends to modulate the gut microbiota by boosting levels of beneficial bacteria. Findings also showed much wider changes in phylogenic composition after GpS treatment in the tumor-bearing than in the healthy nude mice. At this point, the mechanism that accounts for these observations is unclear. Whether this alteration of gut microbiota induced by GpS treatment is linked to the anticancer effect of GpS treatment warrants further investigation. Nevertheless, this study provides fundamental information for understanding how gut microbiota in one mammal changes in response to tumors and to an anti-tumor agent. These findings have broader, significant implications for understanding the role of gut microbiota in both disease and treatment.

## Supporting Information

S1 Fig Quality control of GpS. The GpS contains about 85 to 88% of triterpenoid saponins determined by silica gel thin-layer chromatography (TLC). The ginsenoside Rb1 was used as a titration standard. Each batch of GpS was first generated a UPLC profile, and then compared to the UPLC profile established with 10 pure saponins isolated from the GpS for qualitative control.
(DOC)
