# Supplementary material for: Tumor Grafting Induces Changes of Gut Microbiota in Athymic Nude Mice in the Presence and Absence of Medicinal Gynostemma Saponins
Source: PLoS One. 2015 May 20;10(5):e0126807. doi: 10.1371/journal.pone.0126807 (PMC4439139; doi:10.1371/journal.pone.0126807)
Supplement: S1 Fig — The GpS contains about 85 to 88% of triterpenoid saponins determined by silica gel thin-layer chromatography (TLC). The ginsenoside Rb1 was used as a titration standard. Each batch of GpS was first generated a UPLC profile, and then compared to the UPLC profile established with 10 pure saponins isolated from the GpS for qualitative control. (DOC) [file pone.0126807.s001.doc]

**Figure S1 Quality control of GpS.** The GpS contains about 85 to 88% of triterpenoid saponins determined by silica gel thin-layer chromatography (TLC). The ginsenoside Rb1 was used as a titration standard. Each batch of GpS was first generated a UPLC profile, and then compared to the UPLC profile established with 10 pure saponins isolated from the GpS for qualitative control.
